# ‘Real-world’ priority setting for service improvement in English primary care: a decentred approach

**DOI:** 10.1080/14719037.2021.1942534

**Published:** 2021-06-22

**Authors:** Roman Kislov, Kath Checkland, Paul M. Wilson, Susan J. Howard

**Affiliations:** a Business School, Manchester Metropolitan University, Manchester, UK; b School of Health Sciences, The University of Manchester, Manchester, UK; c National Institute for Health Research Applied Research Collaboration Greater Manchester, Health Innovation Manchester, Manchester, UK

**Keywords:** Priority setting, healthcare networks, decentred governance, primary care, NHS

## Abstract

This article develops an analysis of population-level priority setting informed by Bevir’s decentred theory of governance and drawing on a qualitative study of priority setting for service improvement conducted in the complex multi-layered governance context of English primary care. We show how powerful actors, operating at the meso-level, utilize pluralistic and contradictory elements of complex governance networks to discursively construct, legitimize and enact service improvement priorities. Our analysis highlights the role of situated agency in integrating top-down, bottom-up and horizontal influences on priority setting, which leads to variation in local priorities despite the continuous presence of strong hierarchical influences.

## Introduction

Countries with very different healthcare systems and levels of healthcare spending are grappling with the issue of how to reconcile growing demands and constrained resources. This problem can be addressed by the process of priority setting, which involves ‘decisions about the allocation of resources between the competing claims of different services, different patient groups or different elements of care’ (Klein 2010, 389). Across contexts and levels, population-level priority setting is one of the biggest challenges faced by healthcare decision-makers worldwide (Kapiriri, Norheim, and Martin 2009; Robinson et al. 2012; Hipgrave et al. 2014; Garpenby and Nedlund 2016; Barasa et al. 2017; Petricca et al. 2018). In contrast with bedside rationing where decisions are made about withholding resources from individual patients (Klein 2010; Williams, Dickinson, and Robinson 2011), population-level priority setting focuses on healthcare needs of populations at an aggregate level, with its locus at the level of governments and institutions (Gallagher and Little 2019).

The growth of population-level priority setting is a consequence of neoliberal forms of governance that reproblematize the function of the healthcare system in terms of an economics discourse (Joyce 2001). It is therefore hardly surprising that the extant literature on this subject is predominantly normative and prescriptive. Health economics and the evidence-based movement have been particularly influential in the development of criteria and tools for making formal priority setting decisions (Maynard 1997), with the discipline of ethics offering guidance as to how deliberative processes of priority setting should unfold (Daniels and Sabin 1997). In practice, however, formal, explicit priority setting rarely amounts to more than short-term experiments (Light and Hughes 2001), and most ‘real-world’ priority-setting remains implicit, non-formalized and context dependent (Klein 2010; Cromwell, Peacock, and Mitton 2015; Robinson et al. 2012). However, despite the importance of existing practices for implementation of novel priority setting approaches (Teng, Mitton, and Jennifer 2007), relatively little is known about how narratives of implicit, non-formalized, ‘ad-hoc’ priority setting are constructed and enacted in practice.

To address this gap, this article develops an analysis of population-level priority setting whereby the latter is viewed not as a product of imbalance between supply and demand, but as a linkage between resource allocation, rhetoric and interests of different parties, shaped by a layering of cultural beliefs and social organization (Light and Hughes 2001). It draws on a qualitative study of priority setting for service improvement in primary care conducted in one of the English regions. Service improvement initiatives, aiming to achieve a closer correspondence between actual and desired standards of public services, are usually concerned with the performance of inter-organizational networks (Boyne 2003). We have therefore chosen to explore priority setting in the context of a complex, multi-layered regional network bringing together commissioners (Clinical Commissioning Groups, or CCGs),^1^ operating at meso-level, and providers of healthcare services (general practices), representing the micro-level of governance (Checkland et al. 2018).

Conceptually, our analysis draws on the decentred theory of governance that views policy as a negotiated outcome of diverse and contingent beliefs and actions of situated agents which are shaped by historical traditions and evolve in response to changing situations and dilemmas (Bevir 2011; Bevir and Richards 2009; Bevir and Waring 2020; Bevir and Needham 2017; Bevir and Rhodes 2007). With its focus on the personal agency of actors expressed in narratives and stories (Dickinson 2016), this approach is particularly well suited for exploring the interplay between discursive aspects of real-world priority setting and the features of a broader institutional landscape (Goodwin and Grix 2011). More specifically, our study aims to explore how service improvement priorities are discursively constructed and subsequently enacted in practice against the backdrop of potentially contradictory and inconsistent traditions, policies and rationalities operating in the complex multi-layered context of governance networks.

We describe how powerful actors, predominantly operating at the meso-level of a healthcare network, construct, legitimize and implement priorities for service improvement by creatively incorporating a range of external influences in their situation-specific (and not always internally consistent) priority setting narratives. This leads to a diverse range of priorities adopted for implementation within the same broad geographical area, which partially reflects the differences in population health profiles across its constituent localities but also stems from individual preferences, beliefs and experiences of network actors. These findings show that situated agency, rather than being an undesirable ‘distortion’ (Hofmann 2020), is an integral part of real-world priority setting which is an inherently social, political and discursive process (Smith et al. 2014; Garpenby and Nedlund 2016). They also highlight that opportunities for situated agency are enabled by the complex – and often contradictory – governance arrangements characterizing public sector networks (Jones 2018; Dickinson 2016; Klijn 2012).

This paper is organized as follows. The next section presents three main perspectives (technical, processual and contextual) on priority setting in healthcare, which is followed by discussing priority setting in the context of governance networks. Central concepts of a decentred theory of governance are then introduced and applied to the contextual view of priority setting, within which our own enquiry is embedded. Research setting as well as procedures for data collection and analysis are presented in the Case and Method section. The Findings section describes the following three themes: (1) Factors influencing priority setting at different levels of governance; (2) Translating external influences into priority setting narratives; (3) Enacting commissioners’ priority setting decisions at the level of general practice. The Discussion reflects on these themes in the light of the extant literature. The paper concludes by outlining the main theoretical contributions and practical implications of the study.

## Priority setting in healthcare networks

### Perspectives on priority setting in healthcare

Research on population-level priority setting in healthcare can be classified into three main perspectives: a technical view, a processual view and a contextual view (Klein 2010; Petricca et al. 2018). *The technical view* advocates the development of quantitative tools, such as programme budgeting and marginal analysis (PBMA) and multi-criteria decision analysis (MCDA), to be used for making resource allocation decisions according to formally agreed criteria (Cromwell, Peacock, and Mitton 2015; Mitton et al. 2003). Influenced by health economics, these technocratic solutions decontextualize the benefits of interventions in a way which appears to enable investment decisions to be made comparatively across diverse clinical areas and populations. This approach has been criticized for reducing a complex social process to a mechanistic exercise based on arbitrary metrics, for assuming that this exercise is immune to gaming and participants’ subjective views, and for underestimating the complexity of data and level of decision-makers’ technical competence required to make well-informed decisions (Williams, Dickinson, and Robinson 2011; Holm 1998; Smith et al. 2014). Furthermore, in a public healthcare system which can be viewed as ‘a complex composite of many goals’, all attempts to rationally balance these goals against each other by technical algorithms are destined to remain highly arbitrary (Holm 1998, 1001).

Perceived shortcomings of this perspective have triggered the development of *the processual view* (Holm 1998), which emphasizes procedural justice and fair decision-making processes. Informed by the discipline of ethics, this perspective argues that resource allocation decisions are rife with moral disagreements and a fair, deliberative process is necessary to establish the legitimacy and fairness of such decisions (Robinson et al. 2012). The Accountability for Reasonableness framework has been particularly influential for guiding deliberative priority setting, formulating four conditions – publicity, relevance, revisability and enforcement – as central to transparency and accountability (Daniels and Sabin 1997). Subsequent empirical research into deliberative priority setting has however identified several challenges to its enactment in practice. Implementation of processual approaches can be hindered by epistemological differences, power and status inequalities, poor translation of technical information, limited deliberation and decoupling of the technical and the social forms of knowledge (Crompton et al. 2018). Power differentials limit the inclusiveness of deliberative priority setting (Gibson, Martin, and Singer 2005), and it can still be perceived as unjust due to the failures of knowledge sharing, communication, transparency and mutuality (Gallagher and Little 2019).

Both of these perspectives focus on formal, explicit priority setting and gear towards normative prescriptions about how managers can best manage resources (Smith et al. 2014). However, there is growing evidence that the use of explicit priority setting in healthcare remains compartmentalized and peripheral to resource planning and allocation (Robinson et al. 2012), with resources often allocated on an ad hoc basis (Cromwell, Peacock, and Mitton 2015; Klein 2010) or to satisfy the most people and incur the least opposition (Teng, Mitton, and Jennifer 2007). The latest phase of priority setting research, referred to as the *contextual view*, takes a holistic view of the system by examining the dynamics and interrelationships within it (Petricca et al. 2018). According to this perspective, priority setting requires not only technical but also political knowledge and skills (Garpenby and Nedlund 2016) and is driven by socially defined criteria which are both arbitrary and changeable (Lian 2001). Longstanding structures and organizational relationships can override principles and steps of explicit priority setting, and formally agreed priorities do not necessarily translate into actual changes to resource allocation (Robinson et al. 2012; Hipgrave et al. 2014). Real-world priority setting is influenced by multiple ‘non-technical’ factors (e.g. availability of resources, alignment with the Board’s goals and values, comparability of care offered in other jurisdictions), with organizational and political considerations being as important as health-specific decision-making criteria (Menon, Stafinski, and Martin 2007; Cromwell, Peacock, and Mitton 2015).

### Public sector networks as a context for priority setting

Engaging with the contextual perspective necessitates careful consideration of specific cultural and institutional circumstances in which priority setting is taking place. With the growing demand for integrated service provision, the quality of public service delivery depends on coordinated activities of multiple interconnected organizations (Klijn and Koppenjan 2015; Klijn 2012; Osborne 2010) including, for example, commissioners and providers of healthcare services at different levels. As a result, priority setting for service improvement should be analysed in the context of multi-organizational governance networks rather than single organizations (Boyne 2003). Governance networks are defined as more or less stable patterns of social relationships between mutually dependent but operationally autonomous actors that develop around complex policy issues and contribute to the production of public regulation in the broad sense of the term (Torfing 2012; Klijn 2012).

In this study, we are attentive to the following characteristics of governance networks. Although the network approach to governance emphasizes horizontal coordination, it can still contain strong vertical or asymmetrical elements (Klijn 2012), implying self-regulation in the shadow of hierarchy (Torfing 2012; Héritier and Eckert 2008). Whilst the degree of central influence varies across individual networks (Ferlie et al. 2011) and sectors (Goodwin and Grix 2011), governance networks tend to rely on soft rules rather than hard laws because the network mode of governance is based on negotiation and may lack enforcement mechanisms (Torfing 2012; Klijn et al. 2013). The functioning of governance networks is shaped by a number of external contingencies, such as the clarity or ambiguity of policy, the properties of wider regulatory and institutional context and historical relationships amongst local actors (Waring and Crompton 2020). Finally, whilst the outcomes achieved by governance networks can be influenced by the properties of the network itself (such as size, type of actors involved, goal consensus, resource distribution and quality of relationships) (Varda, Shoup, and Miller 2012; Waring and Crompton 2020), there are also indications that these factors may matter less to the network performance than the strategies of network management (Klijn et al. 2013).

Both the contextual view and the literature on governance networks raise an important question about how key priority setting actors respond to contextual influences and how these responses are mediated by power differentials. On the one hand, in multi-organizational networks no one party can unilaterally and a priori define the nature and quality of public service delivery (Klijn and Koppenjan 2015). That means that through sense-making and framing, leaders have to construct the resource scarcity narratives in ways helping generate and maintain legitimacy and authority needed for implementing priority setting decisions (Dickinson et al. 2011). On the other hand, whether resource allocation decisions are made by politicians, doctors or managers, the typical pattern is that a small group of elite actors rations services for others (Smith et al. 2014; Robinson et al. 2012), which requires political astuteness when dealing with the conflicting values espoused by different stakeholders and creating sufficient consensus to achieve goals (Dickinson et al. 2011; Reeleder et al. 2006).

Given the complexity and variability of factors influencing network management, it is perhaps unsurprising that healthcare literature contains examples of both manipulative network actors advancing their own agendas to the detriment of democratic accountability (Greenaway, Salter, and Hart 2007) and relatively benign post-bureaucratic leadership styles, often taking the form of collective ‘small team based’ leadership (Ferlie et al. 2011, 321). This collective, distributed network leadership involves a high degree of engagement from health professionals drawn into managerial roles, not least due to the fact that shared cultural and institutional structures help clinical leaders enrol colleagues in policy implementation (Oborn, Barrett, and Dawson 2013). To sum up, analysing priority setting in the context of governance networks highlights the complex and nuanced nature of the interplay between multiple influences of inner and outer context, on the one hand, and the situated agency of its key actors, on the other. This will be theoretically explored further in the next section.

## Decentred theory of governance and priority setting

A decentred approach to governance regards networks as constructions of situated agents, whose beliefs and actions are instrumental for creating, sustaining and modifying both policies and institutions. These beliefs and actions are diverse and contingent as they are adopted against the background of multiple (and often competing) historical traditions and evolve in the face of changing situations and dilemmas (Bevir and Richards 2009; Bevir and Waring 2020). Decentred theory rejects positivist accounts of governance that prioritize essential properties of networks, such as size and degree of actor interdependence, or universally operating logics of social or political life (Bevir and Rhodes 2007). It echoes the governmentality literature in recognizing that policy actors draw on historically contingent webs of belief but pays more attention to agency, heterogeneity and variety than reified and monolithic accounts of modern power offered by governmentality theorists (Bevir 2011). Overall, decentred approach emphasizes contingency and context, focusing its attention on how policies are constantly (re)made, (re)negotiated and contested in widely different ways in various everyday practices (Bevir and Waring 2020). The following subsections synthesize key premises of decentred theory of governance with relevant empirical insights to inform the aim and research questions of the study.

### Traditions and dilemmas

In decentred theory, *tradition* is a set of understandings an actor receives during socialization that acts as an underlying influence – but not as a defining force – on subsequent beliefs and actions. Traditions provide a guide to what an actor *might* do rather than rules fixing what they *must* do, which implies that they can be creatively adjusted to novel circumstances (Bevir and Waring 2020). New Public Management – an international ideology aiming to increase the effectiveness of public services through managerial means (Diefenbach 2009) – can be seen as one of the main historical traditions underpinning the rise of priority setting and closely intertwined with the associated technologies of performance management and target-setting. The rise of explicit priority setting also reflects an increasing convergence between New Public Management and the evidence-based movement (Kislov et al. 2019). This intellectual landscape provides a variety of social scientific beliefs and associated technologies to draw on when constructing and implementing healthcare policies (Bevir and Needham 2017).

A *dilemma* arises when a new idea stands in opposition to existing beliefs and practices and forces a reconsideration of these existing beliefs and associated traditions (Bevir and Waring 2020). Traditions change when actors make variations to them in response to specific dilemmas that often stem from perceived failures of governance. As actors confront dilemmas against the background of diverse traditions, there may arise a political contest over proposed solutions, potentially leading to a reform of governance (Bevir and Rhodes 2007). For instance, the network mode of governance, often referred to as New Public Governance, is a relatively new tradition that can be interpreted as an attempt to address the unintended consequences of earlier market-based reforms, such as the fragmentation of the state, by a stronger focus of collaboration and horizontal ties between agencies (Dickinson 2016; Bevir 2011; Osborne 2010; Klijn 2012).

A complex and continuous process of interpretation, contest and modification in response to dilemmas produces ever-changing patterns of governance which are often imbued with contradictions. In practice, the traditions of New Public Management and New Public Governance are closely intertwined, manifesting in hybrid forms characterized by a complex overlay of different governance arrangements (Dickinson 2016; Klijn 2012). In her analysis of the implementation of integrated care in the UK National Health Service (NHS), Jones (2018) shows that distinct policy streams based on networks, hierarchy and markets have accumulated over time, or ‘sedimented’, producing a ‘networked hierarchy’. This hybrid form of governance manifests through setting and monitoring targets, reification of national policy and standardization of local activities. While in some localities hierarchical forms of control are contested and resisted, in others the perceived need to respond to this regime comes to dominate activities of local actors. Markets and formal networks can thus be used by the state as statements of ideology to conceal and bolster central control.

### Narratives and contingencies

When looking at the interplay between traditions, dilemmas and diverse and contingent meanings in action, a decentred approach calls for exploring actors’ *narratives* (Bevir and Rhodes 2007; Bevir and Waring 2020; Waring et al. 2020). These convey complex sets of meanings rooted in historical circumstances and assist actors in confronting novel situations and dilemmas. Narratives provide a framework for meaning-making and social practices and are in turn made and re-made through practice. While a decentred theory acknowledges there have been paradigmatic changes in terms of the dominant modes of governance, such as a shift from hierarchies through markets to networks, these changes are not experienced in the same way by all people (Dickinson 2016). For example, co-operation between commissioners and providers on priority setting can be impeded by clashes of their respective narratives, whereby labelling certain rationing practices as ‘unnecessary or wasteful’ by meso-level decision-makers can potentially antagonize managers and clinicians (Rooshenas et al. 2015). Narratives therefore reflect contingent responses of actors to dilemmas, with different groups often drawing on different traditions to construct markedly different narratives (Bevir and Waring 2020).

In her study of policy implementation in English primary care, Checkland (2018) highlights interdependence between narratives of policy formulation and implementation at different governance levels. At the macro-level, government seeks to reduce resistance by setting conditions in which actors are incentivized to behave in ways consonant with policy objectives, whilst perceiving themselves to be acting autonomously. The resulting absence of clear guidelines leads meso-level actors to come up with a system of shifting and ambiguous rules that only get reified over time. By contrast, in many studies of priority setting conducted in other contexts the central narrative is the lack of autonomy of lower-level authorities from the influence of higher authorities (Petricca et al. 2018; Kapiriri, Norheim, and Martin 2009; Hipgrave et al. 2014; Barasa et al. 2017), whereby the power of local commissioners to lead priority setting is weakened both by the relative strength of national government and clinical opinion (Williams, Dickinson, and Robinson 2011).

As ‘networks are enacted by individuals through the stories they tell one another and cannot be treated as given facts’ (Bevir and Richards 2009, 8–9), applying a decentred approach to priority setting would involve focusing on the social construction – and pluralistic nature – of priority setting narratives. We therefore aim to explore how service improvement priorities are discursively constructed and subsequently enacted in practice through the ability of local agents to create ‘meanings in action’ against the backdrop of potentially contradictory traditions, policies and rationalities operating in a complex multi-layered network context. Our empirical enquiry is guided by the following research questions. *What factors influence priority-setting at the meso- and micro-levels of a healthcare network? How do situated actors translate these influences into formulating priority setting decisions and enacting them in practice?*

## Case and method

### Institutional landscape of English primary care

A number of health systems internationally have embedded priority setting within commissioning functions (Dickinson et al. 2011). The English NHS is no exception.^2^ The government no longer makes explicit decisions about the allocation of resources to different sectors of the NHS (Williams, Dickinson, and Robinson 2011). Instead, resource allocation decisions are delegated to clinical commissioning groups (CCGs) – statutory bodies that have been responsible for purchasing most of health services in the country since 2013. CCGs are comprised of general practices operating within a certain geographical area, reflecting policy-driven transfer of commissioning functions to primary care physicians (Checkland et al. 2013).

The relationship between CCGs and their member practices is complex. As initially constituted, CCGs focus was upon the commissioning of secondary care services, with general practices directly commissioned by NHS England (Checkland et al. 2012). However, subsequently policy delegated to CCGs the responsibility for commissioning primary care services, giving CCGs a role in holding practices to account for the services they provide (McDermott, Checkland, and Coleman 2018). In this complicated, networked context characterized by hybrid accountability relationships (Gore et al. 2019), CCGs hold responsibility for overseeing some aspects of their members’ performance, and for steering some of their activities, whilst practices retain autonomy over day-to-day enactment of their contractual obligations.

This has created a complex institutional landscape when it comes to formulation and implementation of priorities for service improvement in English primary care. CCGs’ governing bodies represent a middle tier of priority setting which is expected to exercise some discretion and responsiveness to local population need while adhering to higher political expectations (Smith et al. 2014). They provide an internationally relevant example of meso-level authorities at the regional level that decide on the mix of programmes, resources and strategies for delivering service improvement interventions (Hipgrave et al. 2014). At the same time, individual general practices, whilst steered by CCGs, largely remain independent businesses providing primary care services to populations and thus, at least in theory, are entitled to determine their own service improvement priorities at the micro-level.

### Data collection and analysis

This study forms part of a broader consultation exercise on priority setting for service improvement which was conducted in 2015 in 13 CCG areas within one of the English regions. The purpose of this consultation was to engage with key commissioners and GPs across all localities within the region in order to understand their service improvement priorities around cardiovascular conditions, identify the support needed to implement these priorities, and inform the development of improvement interventions in these localities. It found no evidence of formal priority setting approaches being adopted across the region, highlighted a significant diversity across different CCG areas in relation to the improvement priorities and needs identified, and concluded that no single improvement intervention was likely to be accepted with equal enthusiasm by all CCGs and/or practices.

This article, aiming to theoretically analyse some of the findings uncovered by the consultation exercise, draws on 45 semi-structured interviews, which were conducted with 23 CCG leads, representing the commissioning (meso-level) perspective on priority setting, and 22 general practitioners (GPs) without commissioning responsibilities, representing the ‘provider’ (micro-level) perspective (Appendix 1). Two overlapping sets of interview questions were developed (one for CCG leads, the other for GPs), accounting for differences in job remits of the two groups. To supplement and triangulate the interviews, we also analysed the content of strategic plans produced by each of the 13 CCGs, with a particular focus on service improvement priorities. Documentary analysis was used for confirming significant diversity of identified priorities across the region but had limited relevance to the resulting empirical account due to lack of detail on how these priorities have been decided.

Data analysis, which was assisted by the NVivo software, unfolded in three phases. The first phase, focusing on the construction of an overarching narrative, combined the codes derived from the interview guide with a set of descriptive codes that emerged inductively. The second phase, utilizing the technique of matrix analysis, focused on mapping similarities and differences between the two groups (Nadin and Cassell 2004). Four research assistants were involved in the process of coding, with a selection of transcripts coded independently and subsequently compared to ensure intercoder reliability. Diversity of priorities, multiplicity of factors driving their selection and the role of key decision makers were identified as promising directions for an in-depth exploration at the third phase. This was led by the first author and was guided by the deductive coding framework informed by the theory of decentred governance and applied to the whole dataset. Codes produced at all the three phases were then combined in an analytical template (King 2004) (Appendix 2). Finally, an iterative process of detecting patterns and developing explanations resulted in the articulation of the three main themes described in the following section.

## Findings

### Factors influencing priority setting at the meso- and micro-levels of governance

As illustrated by Table 1, which systematically presents a selection of interview quotes, both CCG and GP respondents mentioned multiple factors determining what areas of service improvement should be prioritized. It is worth highlighting similarities and differences between the perceived determinants of meso- (CCG) and micro-level (GP) priority setting. First, the role of financial factors was salient at both levels, but they appeared to be framed as ‘cost reduction’ (most often through reducing hospital referrals and admissions) by the CCG respondents and as ‘financial incentives’ (through national or local pay-for-performance schemes) by GPs. The national pay-for-performance incentive scheme, known as the Quality and Outcomes Framework (QOF), was often cited as the most important factor at the level of general practice:

… More often than not, the actual sort of breakdown of QOF into those business groups dictates all of the practice approach to the work in front of them really. (CCG 8.3)

**Table 1.** Factors determining the selection of priorities for service improvement at the Clinical Commissioning Group (CCG) and general practice (GP) level

Level	Factor	Illustrative quote
CCG	National policy	*… If it’s a national agenda and it comes from up above, then yeah it’ll definitely have to be looked into, it’s not a question of whether I personally think it is or not*. (CCG 5.1)
	Population health profile	*Our four priorities are cancer, COPD, mental health and CVD … They are the four biggest killers that we’ve got basically and morbidity as well*. (CCG 12.1)
	Cost reduction	*… We knew initially that our referral rates were very high which was a huge cost burden … We nominated a triager who’s one of the board members of the CCG who now pre-screens the referrals …* (CCG 6.3)
	Audit and performance data	*The starting point … will be CCG priorities … CVD is always on there, COPD is usually on there, dementia has been on there … They’d be long-term areas that don’t benchmark well for varying comparison to other areas, usually*. (CCG 2.1)
	Local integrated care arrangements	*… Within our localities one thing that’s beginning to stimulate interest is the fact that we now have a local dialysis unit in [the area] and we are entering into some discussions with both the providers of the dialysis unit and with [hospital] renal unit … to actually see how we can give a more generic offer around the whole of the kidney agenda* (CCG 7.2)
	Personal interests	*… We rely on each one of our leads really to be aware of any developments in their area, and they will bring things to our monthly meeting and say, look, this bit’s important, this is a new policy, we should do this and then we discuss it* (CCG 8.1)
GP	Priorities set by the CCG	*… The CCG have regular meetings, which I think the practice manager and [one of the GP partners] do go to. I think they often have a set agenda, so I’m not sure why there’s a forum for practices to bring ideas and discuss them … because the agenda is set by the CCG … They have their own priorities …* (GP 13.1)
	Patient experience	*… People can’t get in to see the doctor they want to see and there are big queues outside every morning. So we’re piloting a triage, we triage all the calls and all the patients that want appointments on a Monday morning which, again, it’s the early stages. So, yeah, trying to improve patient access because it’s a constant source of complaint*. (GP 5.2)
	Pay-by-performance scheme (QOF)	*… We pretty much go on the hamster wheel that is QOF every year and just do whatever’s thrown at you for that*. (GP 13.2)
	Other financial incentives	*… Financial incentives always work … It doesn’t have to be big reward, but some recognition of the effort they put in, then I think most practices would respond to that. … It sounds awfully shallow, but at the end of the day … if you’re employing extra staff to do it and things, you can’t do it without money*. (GP 2.1)
	Audit and performance data	*GPs are quite a different breed of people: they like to be compared. … If you have a league table, they want to be towards the top end of the league table*. (GP 13.1)
	Personal interests	*… It’s an area that really interests me, so I wanted to take it on*. (GP 6.1)

Second, as far as the patient-related drivers of priority-setting were concerned, GPs were more attuned to the experiences of individual patients:

… You sit [at the chronic disease meeting] and listen and you get, well, the GPs should do this for this illness, and the GPs should do that for that illness. And you think, how on earth do we do all of that, because then what people forget about is patients come here with their own agenda. (GP 2.1)

By contrast, CCG respondents were more likely to rely on population health data and emphasized the importance of linking the proposed improvement projects to health-related and financial outcomes:

… Being able to demonstrate to [the commissioners] what … some improvement work could do, and showing them the results on a piece of paper and a presentation - that would go a long, long way. … There are other conflicting priorities, but we need to show what the benefit of doing it is between A and B and then showing some outcomes for it … (CCG 4.1)

Third, one of the key findings is the prominence of CCG priorities in the prioritization work of general practices, leaving the latter with relatively few opportunities to shape their service improvement agenda:

… We’ve got the quality improvements set to us by the CCG … It’s decided for us. I mean they do take ideas from practices. However, I can’t help but think that they’re directed from on high then as well as to what the whole … national targets are … . (GP 2.1)

Conversely, at the CCG level, despite some respondents noting that ‘actually you are not setting the priorities, they’re being thrown at you’ (CCG 8.2), there was a much greater recognition of an important translational role of CCGs, combining *top-down* national policy drivers with *bottom-up* information flows concerning local population health profiles and performance outcomes:

… We do now pick things up off national policy, but then we go and have a look at our local data for it as well. If it’s not telling us what the national policy is telling us, then we have a rethink about it. (CCG 4.1)

In addition, *horizontal* information flows relating to secondary and community care organizations located in the same localities also shaped the CCG service improvement agenda, which was often framed as the need to move towards a more integrated provision of services:

… We’ve had an integrated diabetes service for years now, and I’ve worked really closely with the consultant over at [the local hospital] and I wanted to form the same kind of model so that our patients would be getting consistent messages wherever they go … (CCG 8.2)

Finally, although decisions about improvement priorities are made collectively, the influence of powerful clinical leads, with their personal preferences, has been a recurring theme throughout the interviews, particularly at the CCG level:

… It’s a joint group decision … obviously every GP lead wants their disease to be part of that, but we have to come to some consensus between us … (CCG 8.2)

There’s going to be a kind of a national approach to this … but regionally it’s up to you what you want to do. So to me I’ll have to see how I interpret it. (CCG 6.2)

Whilst the selection of priorities was occasionally perceived by the less influential actors as being set in an apparently random, ‘spin-the-bottle’ fashion (CCG 9.2), it is useful to analyse how individual preferences of powerful stakeholders were translated into decision-making. This is the focus of the next subsection.

### Translating external influences into CCG priority setting narratives

Despite the influence of top-down national agenda in CCG-level priority setting, there were several discursive strategies that local powerful actors deployed to rationalize and legitimize their decisions. First of all, there were multiple competing policies at the national level, whereby ‘one priority may be fighting against the other’ (GP 10.1) and ‘if everything is a priority, effectively nothing is a priority’ (GP 5.1). Reflected in the vagueness of CCG’s strategic plans, this plethora of policy choices was further compounded by the multiplicity of criteria used to select projects for prioritization, with the same decision-maker often using different sets of criteria for different improvement priorities:

PAD [peripheral artery disease], I decided because we have had a few limb amputations due to critical limb ischaemia, and I felt that those could have been avoided and prevented *(individual patient experience)*. Then I was a part of the NICE guideline development group of PAD as well, so I wanted to address it ASAP *(special interest in a clinical area supported by belonging to a professional network)*. DVT [deep vein thrombosis], I put that second on the list because we were part of the [regional] DVT pilot project … *(participation in a professional network)*. AF [atrial fibrillation], I put that on the list because of prevalence … (*population health profile)*. … We’ve got poorly managed patients on warfarin and our prescription of new oral anticoagulants is very low as compared to North West and local and national average *(benchmarked audit data)*. Then CKD [chronic kidney disease], I put it there because that was something I was very keen *(personal interest)*. (CCG 7.1; our comments in italics)

In addition, decision-makers creatively switched between two competing co-existing rationalities apparent in the organization of contemporary UK healthcare. On the one hand, there is a more traditional disease-specific discourse (exemplified by the previous quote), in which healthcare delivery in general (and service improvement in particular) is organized along individual clinical conditions or areas of clinical expertise. On the other hand, there is a growing tendency to adopt a more holistic discourse emphasizing the experience of the patient as a whole and promoting the development of integrated care arrangements working across multiple clinical disciplines and sectors:

… It’s interesting how we’ve moved away from focusing so much on specific areas, I think last year we had a focus on COPD [chronic obstructive pulmonary disease], and we’ve had some clinical focus on atrial fibrillation as well. And a lot of the focus is now put into … things that result in multiple conditions, as if we can affect the whole by dealing with it as a whole as opposed to dealing with individual pieces … (CCG 9.2)

The most important priorities … are more structural and organizational … It’s about more integrated system wide working across general practice, community services, hospital services, mental health services and social care. So that’s more the focus of where we’re looking at the moment than specific clinical conditions. (CCG 10.1)

Co-existence of these rationalities further increased the number of discursive choices available to decision-makers to rationalize their personal preferences, whereby the same improvement project could be prioritized in one CCG and deprioritized in the other:

… Long term conditions, cardiovascular is my area of work and I’ve become quite passionate about kidney health over the years. So I say that kidney health runs through diabetes, runs through cardiology, cardiovascular. So if we address kidney health it will address the other areas of work as well. So it’s kind of incorporated into other specialities rather than a speciality by itself. (CCG 6.2)

I mean CKD [chronic kidney disease], yeah absolutely it’s one LTD [long-term disease], but there is multiple of those and we all know that individuals, especially the frail elderly, they will have multiple co-morbidities at one time. So we’ll have to ensure that emphasis on a particular disease parameter is … there’s a balance to it. You know, why CKD, why not diabetes for example? (CCG 5.1)

The strategies described above further emphasize the inherently arbitrary and contestable nature of priority setting at the CCG level. The next subsection will explore how these priorities were translated into the priorities of general practices, over which CCGs had relatively limited direct control.

### Enacting commissioners’ priority setting decisions at the level of general practice

The most obvious strategy used by CCGs was aligning their improvement priorities with the requirements of existing pay-for-performance schemes such as QOF, as these represented a significant proportion of general practice income. In those cases where the CCGs wanted to stimulate improvement above the existing QOF standards or where relevant QOF indicators were not available, additional financial incentivization schemes, tied to appropriate performance indicators, had to be introduced to enable implementation of priorities at the level of general practice:

… QOF incentivizes you to get to a certain target. So say it’s 90% for whatever … For practices once you’ve got to your 90% or whatever the target might be … you’ve then got to go and move to the next phase because again as a practice they’ve got to do everything … What the [local incentivization scheme] should do is pick up that 10%. (CCG 12.1)

… Those [indicators] have been removed, [but] we want practices to still do them … because we still think those indicators are markers of good care, so we’ve asked practices to carry on doing those things … so we will incentivise people still doing all those things that they’ve always been doing. (CCG 8.1)

CCGs in our sample differed as to the breadth of incentivization offered. Some invited their practices to bid for additional funding or promoted specialization of certain practices in selected clinical areas, enabling the best-performing practices not only to resource their own service improvement agenda but also shape the priorities of the CCG area as a whole:

… We’ve said that one practice has got to take the lead on behalf of the cluster for managing the implementation and the commissioning of those services. (CCG 7.3)

We’ve got a fund … and we invite people to make a bid for something like a pilot … So someone [from a general practice] would come and present a bid, we’d have a look at it, we’d make amendments and then … if it’s good work, we’ll give it to somebody to try for a year and then report back to us and if it worked really well, we might say, oh this is something that we want to commission long term … (CCG 8.1)

Others were critical of selective attention to pilot projects and positive outliers and advocated for a blanket approach, applying a relatively limited set of incentivized service improvement standards to all practices under their jurisdiction:

… In most other CCGs it was just pockets of excellence where a number of practices took part [in an improvement project], and the majority never did, so it didn’t really contribute to overall outcomes, while in [our two areas] it was rolled out across … I’m not very keen on pilots. Pilots get you volunteer practices that participate enthusiastically. It makes not one jot of difference to population health because it’s not big enough. You have to get every practice involved. … I’ve written a [local incentivized standard], that’s around 19 areas of work and 40 KPIs which every practice is participating in. I’m investing an extra 3.4 million pounds into primary care, so that they might achieve these standards … (CCG 1.2)

This quote highlights the importance of audit and performance standards in general practice, and many CCGs used the embeddedness of benchmarking in the primary care culture as a basis for another strategy deployed to translate CCG priorities to the level of general practices. This strategy entailed identifying practices whose performance indicators in priority areas were worse than those of their peers and targeting them for relevant service improvement projects:

So each practice might have different areas that they want to improve on … You might find one practice, for example, needs to look at their prescribing of benzodiazepines, whereas another practice needs to look at their ear, nose and throat referrals, whereas another practice needs to look at their diagnosis of COPD, whereas another practice needs to look at their treatment of CKD patients. (CCG 11.2)

… We usually from the CCG get a list of things and they say, pick three of these. … And it’s usually tied in to how well we’re doing … So they will usually say, well, look, you need to do some of the ones where you’re doing worse compared to everyone else, rather than the things you’re doing well at. (GP 13.1)

## Discussion

**Figure 1.**
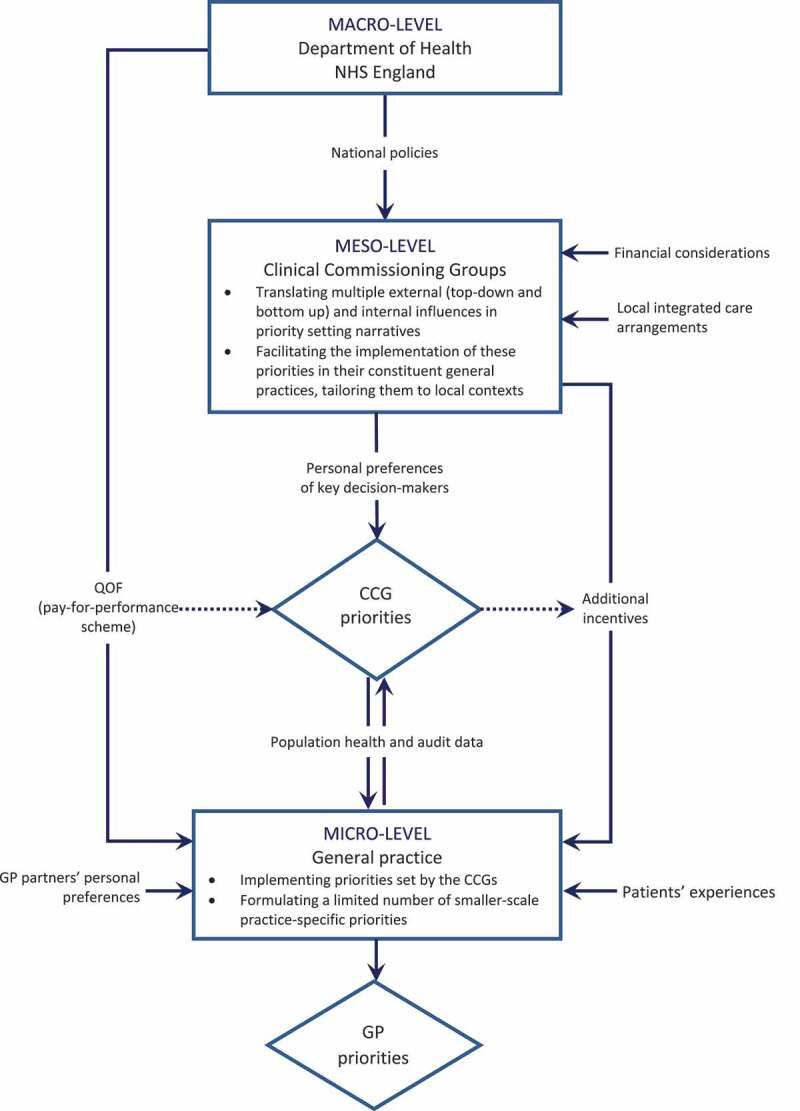
Priority setting in a multi-layered governance context

We have presented real-world priority setting for service improvement as a process taking place in the context of a complex multi-layered governance network. Previous research suggests that in the absence of formally agreed criteria, complex and interconnected structures of healthcare governance networks may constrain opportunities for lower-level authorities to reallocate resources, and that implicit priority setting may be influenced by historic patterns of allocation that become institutionalized, restricting viable future priority setting options (Smith et al. 2014). Our findings, however, point towards a more nuanced picture (Figure 1). On the one hand, real-world priority setting in primary care is indeed shaped (and often significantly constrained) by multiple political and institutional factors operating at different levels of governance. On the other hand, decentralizing influences are also apparent. These most often take the form of locally collected population health data reflecting cross-area differences in population health profiles and informing bottom-up knowledge flows between the micro- and meso-levels of governance. In addition, there are also horizontal influences, for example considerations given by CCGs decision-makers to their local links with secondary care providers and other integrated care arrangements within their localities.

But how do these vertical and horizontal influences translate into the formulation and enactment of actual priorities for service improvement? We have addressed this question using the decentred theory of governance which highlights the interplay between the narratives of network actors and the complex socio-political context in which they are embedded (Goodwin and Grix 2011). It is exactly this complex, multi-layered landscape that enables CCGs, as meso-level agents of priority setting, to construct and enact narratives of priority setting bringing together national policies, regional integrated care landscapes and divergent local interests of general practices. Furthermore, rather than seeing historical patterns constrain priority setting at all times (Teng, Mitton, and Jennifer 2007; Smith et al. 2014), this study suggests that these patterns may also contain dilemmas and contradictions that can be deployed by network actors to their advantage. What results is an emerging variation in local priorities despite the continuous presence of strong hierarchical influences.

By describing this process, this study highlights how different groups of contextual factors – as well as opposing underlying policies and rationalities (such as the disease-focused paradigm and the service integration paradigm) – can be selectively deployed by the same actor depending on the priority setting narrative they are trying to construct, often reflecting their own personal agenda. Interestingly, the development of narratives switching between competing policies and rationalities did not seem to be accompanied by the experiences that could be interpreted as ‘dilemmas’ as defined in the classic formulations of decentred theory, i.e. as a clash between the dominant tradition and perceptions of actors, leading to modification or rejection of this tradition (Bevir and Rhodes 2007). In our case, dilemmas manifested not as confrontations between new ideas and existing practices (Bevir and Waring 2020) but as highly contingent situation-specific choices between different competing policies and rationalities on offer. Most importantly, our findings suggest that posing and resolving such dilemmas may help situated actors incrementally advance their agendas without triggering major change, disrupting the status quo or resolving underlying contradictions or inconsistencies.

These contradictions and inconsistencies stem from hybrid, sedimented nature of governance traditions characterizing contemporary policy context of Anglo-Saxon countries and combining hierarchies, markets and networks (Dickinson 2016; Jones 2018). On the one hand, absence of explicit, formalized priority setting approaches (such as the ones described in the literature review as ‘technical’ or ‘processual’) in the region we examined can, for instance, be explained by the mismatch between the underlying assumptions of formal priority setting, which arose in response to New Public Management and the evidence-based movement, and the network aspects of English primary care context shaped by New Public Governance. At the same time, our findings also suggest that contradictions between different layers of traditions constituting sedimented governance become embedded in policy ‘master frames’ and therefore taken for granted by network actors, who creatively incorporate them in their individual narratives (‘local action frames’) of implicit priority setting (cf. Torfing 2012; Waring et al. 2020). This helps explain why the latter may end up being inconsistent in themselves and at odds with one another.

Similar inconsistencies and tensions are capitalized on when service improvement priorities previously agreed at a meso-level are enacted at the lower, micro-level of governance. Reinforcement of existing mechanisms of *hierarchical control* is apparent in the alignment of prioritized service improvement projects with relevant QOF indicators or other centrally set targets. *Market-based mechanisms* are in operation when CCGs devise additional incentive schemes for supporting the implementation of those improvement priorities that are not covered by the QOF. And *network mechanisms* are activated when soft power of benchmarking is used in CCGs’ dealings with negative outliers, reflecting GPs’ competitiveness as a fundamental cultural characteristic of English primary care as a multi-layered network (Kislov, Hyde, and McDonald 2017; McDonald 2018). Translation of meanings into action in meso-level decentred governance requires rhetorical strategies to be coupled with the more tangible forms of resources. This significantly narrows down the number of priority setting drivers viewed as important at the enactment stage, potentially limiting the breadth of options available to meso-level network actors in terms of seeing their priorities implemented in practice. However, these constraints are partially offset by variability in how broadly incentivization and benchmarking can be applied to constituent GP practices. As a result, some of the enactment strategies, such as selecting positive outliers for pilot projects or targeting negative outliers, may lead to further divergence of service improvement priorities not only across CCGs but also within the same CCG area.

Our findings suggest that the role of individual beliefs, experiences and preferences of meso-level network actors in real-world priority setting is generally seen as legitimate and remains unchallenged despite some differences in framing apparent between CCGs and general practices (cf. Rooshenas et al. 2015). This can be explained by a combination of factors. First, these network actors use their ‘political astuteness’ (Dickinson et al. 2011) or ‘craftsmanship’ (Bianchi, Nasi, and Rivenbark 2021) to interweave various political, financial, clinical and organizational factors in order to justify the selection of certain areas, programmes or projects of work for prioritization. Second, fluidity, malleability and internal inconsistency of narrative frames deployed by the actors may be an indication that these frames are not as deeply rooted in the actors' underlying core beliefs as would be expected in the situation of a significant network conflict (Klijn and Koppenjan 2015) or political contest (Bevir and Rhodes 2007). Finally, legitimation of these elite actors is not limited to purely discursive factors. In addition to the strategies of enactment described above, it also involves recognition of these actors’ formal status in the governance networks which, in turn, stems from their expertise in certain clinical areas (cultural capital) and their connections with other powerful actors within and beyond the primary care network (social capital), such as hospital consultants and government arm’s-length agencies. These findings extend previous observations about multiple sources of legitimation for actors operating in inter-organizational networks (Kislov, Hyde, and McDonald 2017; McDonald 2018) by highlighting the complex interplay between legitimation discourses and different forms of capital in enacting change.

Decentred theory emphasizes radical contingency generated by diverse beliefs and practices of situated agents and does not aim to identify general, uniform, universally applicable accounts of governance processes. It is, however, important to highlight structural and institutional factors that may have contributed to the patterns of priority setting uncovered by this study (Goodwin and Grix 2011). First, *characteristics of the network’s constituent organizations* matter. CCGs are clinically led organizations by relatively high levels of professional discretion which promotes situated agency (Wright and Turner 2021). Second, *relatively broad scope of the network* implies a broader pool of underlying inconsistencies and contradictions creatively deployed by local actors, compared to more topic-specific ‘managed’ networks, such as regional networks for cancer (Addicott, McGivern, and Ferlie 2007) or health and social care integration (Jones 2018), which have been shown to be more amenable to central control. Finally, situated agency of meso-level network can be enabled by a *relatively high degree of network fragmentation*. In UK commissioning frameworks tend to be relatively decentralized and light-touch in terms of regulation (Bovaird, Briggs, and Willis 2014), with minimal unifying governance structures, ambiguous hierarchies and emphasis on local contingencies in favour best practice as features of the institutional landscape of English primary care (Gore et al. 2019). Such context is favourable for development of policy entrepreneurship, but multiple, albeit not always clearly delineated, upstream, downstream and horizontal accountabilities (Ferlie et al. 2017) provide checks against excesses of situated agency of meso-level network actors.^3^

### Limitations

Situated agency and resulting variability of priorities observed in our case are enabled by the relatively pluralistic, multi-layered and professionally led governance context described above. Our conclusions may therefore be less applicable to more hierarchical and centralized networks (Addicott, McGivern, and Ferlie 2007) or those governance structures that involve direct democratic election of representatives (Ferlie et al. 2017; Wolf and Bryan 2009). Our findings about the actual drivers, narratives and practices of priority setting decisions are derived from interviews and, although triangulation was conducted both across multiple accounts and across two methods of data collection, our conclusions are limited by the lack of observational data. On the other hand, due to our explicit interest in narratives of priority setting, it can be argued that interviews were the most appropriate method of data collection in our case (Lian 2001). Finally, whilst we acknowledge the consequences of population-level priority setting for bedside rationing, exploring the interrelationship between the two lay outside the scope of this article and could be a fruitful avenue for future research on priority setting, along with studying ways of incorporating the views of patients and the public in the process of resource allocation.

## Conclusion

This paper advances the contextual view of priority setting, addressing the call for building an empirical foundation of implicit priority setting (Martin and Singer 2003) to explore the complex interplay between macro- and meso-level structures, on the one hand, and situated agents and their discourses, on the other (McDonald 2018). Our theoretical contribution is threefold. First, we enhance understanding of how discourses and storylines shape interaction between actors in governance networks (Torfing 2012) by describing how meso-level decision-makers discursively deploy tensions and contradictions between different traditions, policies and rationalities to construct situation-specific, even if not always internally consistent, narratives of priority setting. Second, we demonstrate how these priority setting narratives are enacted in practice, going beyond mere ‘communication of decisions to stakeholders’ described in the existing processual models of priority setting (Menon, Stafinski, and Martin 2007) and thus addressing the call to explore the challenges of downstream implementation in post-New Public Management forms of governance (Sørensen and Torfing 2021). Finally, we highlight that diverse beliefs, experiences and interests of meso-level actors, coupled with enabling structural factors, lead to a significant variation in service improvement priorities, with this analysis extending our understanding of the discretionary power of lower-level actors in governance networks (Hughes and Griffiths 1999).

This work raises several practical implications for those involved in designing, implementing and evaluating priority setting initiatives in network contexts. First, rather than labelling key decision-makers’ framing and legitimating activities as ‘biases’ hindering priority-setting (Hofmann 2020), these contributions should be explicitly acknowledged and discussed as inherent to any priority-setting exercise, with a greater awareness of both their positive and negative consequences. Second, uptake of service improvement priorities in healthcare networks can be maximized if these projects make explicit connections with multiple priority-setting drivers, ensure support of key decision-makers at meso-level, and consider mechanisms through which implementation at the micro-level of healthcare providers will be incentivized and resourced. Finally, collaborative priority setting needs to pay attention not only to bridging differences in frames and discourses *among* actors (Klijn and Koppenjan 2015) but also to promote critical self-reflection targeting inconsistencies and contradictions *within* individual narratives.

## Data Availability

The data that support the findings of this study are available on request from the corresponding author. The data are not publicly available due to privacy restrictions.

## Notes

1. In the UK context, commissioning refers to procuring services from providers, ensuring that they meet health and social care needs of local communities (Bovaird, Briggs, and Willis 2014).

2. The NHSs in Scotland, Wales and Northern Ireland do not follow this model as they are governed by devolved administrations.

3. Although the COVID-19 pandemic may have had a centralizing effect on some health and social care networks (Yang 2020), the recent UK White Paper (Department of Health and Social Care 2021) suggests that NHS is moving towards an even more networked form, with an increased ambiguity of hierarchies and accountabilities, which potentially makes our findings as pertinent now as they were at the time of data collection and analysis.

## Appendix 1. Research sample

**Table:**

	CCG respondents	GP respondents	*Total*
Area 1	2	2	*4*
Area 2	2	3	*5*
Area 3	1	3	*4*
Area 4	1	1	*2*
Area 5	1	2	*3*
Area 6	3	3	*6*
Area 7	3	0	*3*
Area 8	4	2	*6*
Area 9	1	1	*2*
Area 10	1	2	*3*
Area 11	2	0	*2*
Area 12	1	1	*2*
Area 13	1	2	*3*
*Total*	*23*	*22*	*45*

We contacted 156 practices to ensure that at least one general practitioner (GP) is interviewed in each of the Clinical Commissioning Group (CCG) areas, but only 22 practices agreed to take part in the study. That explains why we were unable to recruit any GP research participants in two CCG areas.

## Appendix 2. Final coding template

1. **Factors influencing priority setting**
  1. National policy
    1. Integrated care
    2. Other policy drivers
  2. Population health profile
  3. Cost reduction
  4. Audit and performance data
  5. Local integrated care arrangements
  6. Personal interests
  7. Priorities set by the Clinical Commissioning Group (CCG)
  8. Financial drivers
    1. Quality and Outcomes Framework (QOF)
    2. Other financial schemes
  9. Patient experience
2. **Translating external influences into CCG priority setting narratives**
  1. Traditions/rationalities
    1. Disease-specific approach
    2. Holistic approach
  2. Dilemmas/tensions
    1. Competing national policies
    2. Competing traditions/rationalities
  3. Narratives/discursive strategies
    1. Switching between different criteria/drivers of priority setting
    2. Switching between different traditions/rationalities
3. **Enacting CCG priority setting decisions at the level of general practice**
  1. Incentivization
    1. Blanket approach
    2. Selective approach
      1. Bidding
      2. Specialization
  2. Benchmarking
    1. Between CCG areas
    2. Between general practices within the same CCG
4. **English primary care as institutional context**
  1. NHS England
  2. Priority setting forums
    1. CCG meetings
    2. General practice meetings
  3. Direction of network governance
    1. Top-down
    2. Bottom-up
    3. Horizontal
